# High-dose ascorbic acid synergizes with anti-PD1 therapy in non-small cell lung cancer *in vitro* and *in vivo* models

**DOI:** 10.3389/fimmu.2024.1512605

**Published:** 2025-01-17

**Authors:** Hak Su Kim, Seung-hyun Kwon, Ok Kyung Choi, Taekyu Lim

**Affiliations:** ^1^ Veterans Medical Research Institute, Veterans Health Service Medical Center, Seoul, Republic of Korea; ^2^ Division of Hematology-Oncology, Department of Internal Medicine, Veterans Health Service Medical Center, Seoul, Republic of Korea

**Keywords:** anti-PD1, high-dose ascorbic acid, non-small cell lung cancer, immune checkpoint inhibitors, proteomic analysis

## Abstract

**Introduction:**

Immune checkpoint inhibitors(ICIs) targeting programmed cell death protein 1 (PD1) confer significant survival benefits to patients with non-small cell lung cancer (NSCLC). However, there remains a substantial unmet need to identify therapeutic approaches to overcome resistance and provide benefits to these patients. High-dose ascorbic acid (AA) acts synergistically with many standard anticancer treatments. However, little is known about the effect of high-dose AA on improving the efficacy of anti-PD1 inhibitors in NSCLC. This study aimed to elucidate the effects of high-dose AA on anti-PD1 immunotherapy in NSCLC.

**Methods:**

The combined effects of high-dose AA and anti-PD1 were investigated using a coculture model of H460 cells and CD8+ T cells and an LLC1 lung cancer syngeneic mouse model. To investigate the molecular mechanism, tumor tissues from mice were analyzed by comprehensive proteomic profiling using nano-LC-ESI-MS/MS.

**Results:**

Pretreatment with a high dose of AA led to enhanced the sensitivity to the cytotoxicity of CD8+ T cells derived from healthy donor for H460 cells. Additionally, the combination of anti-PD1 and high-dose AA significantly increased CD8+ T cell cytotoxicity in H460 cells. The combination of anti-PD1 and high-dose AA showed dramatic antitumor effects in a syngeneic mouse model of lung cancer by significantly reducing tumor growth and increasing CD8+ T cell-dependent cytotoxicity and macrophage activity. Comprehensive protein analysis confirmed that high-dose AA in anti-PD1-treated tumor tissues enhanced the antitumor effects by regulating various immune-related mechanisms, including the B cell and T cell receptor signaling pathways, Fc gamma R-mediated phagocytosis, and natural killer (NK) cell-mediated cytotoxicity.

**Discussion:**

Our results suggest that high-dose AA may be a promising adjuvant to potentiate the efficacy of anti-PD1 immunotherapy.

## 1 Introduction

Lung cancer is the leading cause of cancer-related deaths worldwide, with non-small cell lung cancer (NSCLC) accounting for more than 80% of cases (1). Immune checkpoint inhibitors (ICIs), such as anti-programmed cell death 1 (anti-PD1) and anti-programmed cell death ligand 1 (anti-PD-L1) antibodies, have changed the treatment paradigm for NSCLC, which lacks actionable genomic alterations, both as monotherapy and combination therapies. Although long-term survival rates after treatment with ICI were significantly higher than previous treatments without ICI, only a small proportion of patients (less than 30% of patients) experienced benefits (2). Moreover, in some cases, immune-related adverse events (IrAEs) limit the efficacy of ICI treatment.

Ascorbic acid (AA), also known as vitamin C, is an essential water-soluble micronutrient with a six-carbon structure (C_6_H_8_O_6_) that plays vital roles in many biological processes. Humans rely solely on dietary intake for AA, due to mutations in the gene that produces L-gulono-γ-lactone oxidase (GLO), which is an major enzyme catalyzing the final step of AA formation (3, 4). AA is a potent antioxidant protecting the body against endogenous and exogenous oxidative challenges. In addition, it is a cofactor for numerous biosynthetic enzymes, and its gene regulatory function plays a crucial role in its immune-modulating effects (5).

Several studies examining the therapeutic effects of AA on cancer have found that AA at pharmacological concentrations from intravenous administration, but not physiological AA from oral ingestion, exerted clinical benefits in patients with cancer (6–8). Optimal levels of AA levels may be clinically important in cancer, and observational studies support an inverse correlation between circulating AA and cancer (9, 10). A recent study demonstrated that average plasma AA levels in patients with more advanced cancer who were receiving chemo or immunotherapy were lower than those in the presurgical cohort. Patients currently undergoing chemotherapy or immunotherapy may be at a particular risk of AA depletion associated with increased requirements and not necessarily due to reduced intake (11).

Over the past few decades, several studies have focused on the role of AA in the human immune response. The maturation, proliferation, and viability of T cells, which actively acquire AA via sodium-dependent vitamin C transporters (SVCT) and sodium-independent glucose transporters (GLUT) and have intracellular AA concentrations 10–100-fold higher than plasma levels, are upregulated by the presence of normal physiological concentrations of AA (12, 13). High-dose AA synergizes with anti-PD1 and anti-cytotoxic T lymphocyte associated protein 4 (anti-CTL-4) in mouse models, and increases the immunogenicity of effector T cells in many cancer types, including breast, colorectal, melanoma, pancreatic cancer, and lymphoma (14, 15). Recently, Zhao et al. demonstrated that high-dose AA inhibited PD-L1 expression in breast cancer cell lines, enhanced antitumor effects of T cells, and inhibited PD-L1 transcription through the ROS-phosphorylated signal transducer and activator of transcription 3 (pSTAT3) signaling pathway (16).

The development of rational and safe combination therapies to improve the efficacy of ICIs remains an unmet need. In addition, experimental studies on the effects of high-dose AA in improving the sensitivity of NSCLC to ICIs are lacking.

In this study, we hypothesized that high-dose AA synergizes with anti-PD1 therapy in NSCLC. We investigated the antitumor effects of high-dose AA in combination with an anti-PD1 antibody in lung cancer cells *in vitro* and *in vivo*. The molecular mechanism of high-dose AA was analyzed through a comprehensive proteomic profiling technique using liquid chromatography with tandem mass spectrometry (LC-MS/MS).

## 2 Materials and methods

### 2.1 Ethics statement

This study was completed under Protocol, which was approved by the Institutional Review Board of VHS Medical Center, Seoul, Republic of Korea (approval no.2022-07-004). The mouse experiments were performed in accordance with the Guiding Principles for the Care and Use of Animals. All protocols were approved by the Animal Care and Handling Committee of Kyung Hee University Medical Center (protocol #KHNMC AP 2023-04).

### 2.2 Cell culture

Human lung cancer cell line H460 was used in this study. LLC1 (mouse Lewis lung carcinoma; American Type Culture Collection, ATCC) was used to establish a lung cancer syngeneic mouse model. H460 was cultured in RPMI1640 (Hyclone) supplemented with 10%FBS and 1% penicillin/streptomycin. LLC1 was obtained from the ATCC and was maintained in Dulbecco’s Modified Eagle’s medium supplemented with 10% FBS and 1% penicillin/streptomycin. Most cells were grown to approximately 70% confluence in culture dishes at 37°C in a 5% CO_2_ incubator, and detached using 0.25% trypsin EDTA for passaging or use. T cells were isolated from peripheral blood mononuclear cells (PBMCs) by negative isolation (Dynabeads™ Untouched™ Human CD8 T Cells Kit 11348D; Thermo Fisher) and cultured in RPMI-1640 medium with 2mM L-Glutamine (Gibco), 10% FBS and 100U/mL penicillin/streptomycin. For activation of CD8+ T cells, isolated CD8 T cells were cultured in the presence of anti CD3/CD28 beads (Dynabeads™ Human T-Activator CD3/CD28 11131D; Thermo Fisher). Two days after isolation, CD8+ T cells were used in experiments.

### 2.3 *In vitro* AA treatment

For cell culture experiments, we used a stock solution of 0.5 M L-ascorbic acid (255564; Sigma-Aldrich) in phosphate buffered saline (PBS). The pH of the culture medium was not significantly altered by treatment with 1 mM AA. High-dose AA induces cytotoxicity in cancer cells via various mechanisms such as redox imbalance, epigenetic regulation, and immunomodulation (17). Prooxidant function of AA targeting redox imbalance has been most widely described as a cytotoxic mechanism (18, 19). To catalase to protect H_2_O_2_ induced cytotoxicity, CD8+ T cells and H460 cells were pretreated with 1000 units/ml catalase (60634; Sigma) for 30 min (14, 20). Cells were treatment with or without 1 mM AA (Sigma) for 6 h. Both catalase and AA were prepared fresh for each experiment. After 6 h of treatment, the cells were washed and fresh medium was added. Cells were harvested for downstream analyses 18–24 h after treatment.

### 2.4 Cytotoxicity assay

Cytotoxicity was assessed using lactate dehydrogenase (LDH) assay. CD8+ T cells derived from healthy donor or H460 cells were pretreated with 100 µg/ml catalase for 30 min and then exposed to the indicated concentration of AA for 6 h. Cells were resuspended in fresh medium and incubated for 24 h or 48 h. Cells treated with lysis buffer supplied from Quanti-LDH cytotoxicity assay kit (BIOMAX, Seoul, South Korea) were acted as maximum LDH activity controls. Culture medium (10 µL) was mixed with 100 µL of the reaction mixture at room temperature for 30 min in the dark. Subsequently, 10 µL stop solution was added. The absorbance was measured at 450 and 650 nm.

### 2.5 Syngeneic animal model

Seven-week-old female C57BL/6J mice were obtained from Orient Bio (Seongnam-si, Gyeonggi-do, South Korea) and acclimatized for one week before the experiments. Mice were housed under specific pathogen-free conditions in an air conditioned (22 ± 2°C) and humidity-controlled (45–55%) room under a 12-h light and 12-h dark cycle with *ad libitum* access to food and water. 1x10^6^ LLC1 mouse lung cancer cells in matrigel were injected subcutaneously into the right abdominal region. After seven days, when the tumor masses reached ~130 mm^3^, the mice were randomly divided into four groups: 1) vehicle plus isotype control (n=5), 2) vehicle plus anti-PD1 (n=5), 3) AA plus isotype control (n=6), and 4) AA plus anti-PD1 (n=6). 300-μL intraperitoneal (i.p.) injections of 1.5 M ascorbate or NaCl were given daily. To balance the osmotic effect of sodium L-ascorbate, the mice that did not receive ascorbate (vehicle or anti-PD1) were administered NaCl (Sigma-Aldrich). Added to these i.p. injections every other day was 200 μg anti-PD1 (BE0146; BioXCell) (anti-PD1 and AA+anti-PD1 groups) or isotype control (BE0089; BioXCell) (vehicle and AA groups) beginning on the first day of treatment, tumor volume and mouse weight were monitored every 3 days for 3 weeks. Tumor volume was evaluated in accordance with the formula (*L* х *l* ^2^)/2 through the measurement of tumor length (*L*) and width (*l*) using calipers. The mice were then sacrificed, and their tumors and blood PBMC were harvested on day 21. Tumor tissues from mice were divided into two parts, the one was fixed at 4% paraformaldehyde for immunofluorescence analysis, and the other was stored at −80°C for Proteomic analysis. The coefficient of drug interaction (CDI) was used to calculate the synergy with the combination using mean tumor volume measurements and was evaluated in accordance with the formula AB/(A × B), where AB is the ratio of the 2 drugs combination group to the control group, and A or B is the ratio of the single drug group to the control group. CDI < 1 indicates synergism, CDI = 1 indicates an additive effect, CDI > 1 indicates antagonism, and CDI<0.7 indicates a significant synergistic effect.

### 2.6 Immunofluorescences staining

Tumor tissues were fixed in 4% formaldehyde and embedded in paraffin. Each sample was processed and embedded in its own cassette. paraffin-embedded blocks were sectioned at 4 μm onto positively charged slides. Antigen retrieval was performed using Leica Bond Epitope Retrieval Buffer 2 for 20 min. Non-specific background was blocked with 10% goat serum for 1 h. Each tissue was stained with primary antibodies against CD8 (14-0808-82-rat; Thermo Fisher, MA, USA), granzyme B (AF1865-Goat; R&D Systems, MN, USA), F4/80 (ab111101-rabbit; Abcam, CB, UK), CD11c (CSB-PA011879ESR1HU-rabbit; CUSABIO, TX, USA), and IL-12 (ab131039-rabbit; Abcam, CB, UK) for 60 min. Secondary antibodies including anti-rat (A21208-Alexa Fluor™ 488, Thermo Fisher, MA, USA), anti-rabbit (A-31573-Alexa Fluor™ 647, Thermo Fisher, MA, USA), and anti-goat (A21447 Alexa Fluor™ 647, Thermo Fisher, MA, USA) were incubated for 60 min. Slides were mounted with DAPI for nuclear visualization. Whole-slide images were obtained using a confocal microscope (FV3000RS; Olympus). % area was quantified by image J software.

### 2.7 Nano-LC-ESI-MS/MS analysis

Each tumor tissue from mice dissolved in 100 µL of 5% SDS buffer was treated with 20 mM dithiothreitol in 50 mM ammonium bicarbonate, reduced for 10 minutes at 95°C, and then alkylated with 40 mM iodoacetamide in 50 mM ammonium bicarbonate for 30 minutes in darkness. Proteomic samples were prepared using S-TRAP™ (Protifi, Farmingdale, NY, USA) according to the manufacturer’s protocol and a previous study (21). Each sample was incubated with 12.5 µg of sequencing-grade modified trypsin/LysC (Promega, Madison, Wisconsin, USA) in a 50 mM ammonium bicarbonate solution (pH 7.8) using an S-TRAP column overnight at 37°C. The eluted peptide samples were then dried and quantified. The samples were resuspended in 0.1% formic acid and analyzed using an UltiMate 3000 RSLCnano system coupled to a Q Exactive Plus Hybrid Quadrupole-Orbitrap mass spectrometer with a Nano-ESI source (Thermo Fisher Scientific), according previous study (22). Tryptic peptides separated on an Acclaim™ Pepmap 100 C18 column (500 mm × 75 μm i.d., 3 μm, 100 Å) equipped with a C18 Pepmap trap column (20 mm × 100 μm i.d., 5 μm, 100 Å; Thermo Fisher Scientific) over 200 min (250 nL/min) using a 5–40% ACN gradient in 0.1% formic acid and 5% dimthyl sulfoxide for 150 min (250 nL/min) at 50°C. Mass spectra were acquired in the data-dependent mode with an automatic switch between the full scan and the top 20 data-dependent MS/MS scans. The target value for the full-scan MS spectra, selected from 350 to 1800 *m*/*z*, was 3,000,000 ACS targets with a maximum injection time of 100 ms and a resolution of 70,000.

### 2.8 Data search, statistical analysis, and bioinformatics analysis

The MS/MS spectra were assigned to proteins using Sequest-HT on Proteome Discoverer (Version 2.4, Thermo Fisher Scientific) and the UniProt mouse database (22). The identified proteins were analyzed and visualized using Perseus software (Version 2.0.7.0). One-way analysis of variance (ANOVA) with Benjamini-Hochberg method-based false discovery rate (FDR) and a significance level of 0.05 were used to identify significant differences in the protein expression levels. Gene Ontology (GO) annotation of the proteins identified from the proteome analysis was performed using Proteome Discoverer (Version 2.4; Thermo Fisher Scientific). A protein-protein interaction (PPI) network was constructed using the STRING website (www.String-db.org). Statistical analysis of the expression levels of selected individual proteins was performed using GraphPad Prism software (version 5.0). Significant differences were analyzed using one-way ANOVA followed by the Newman-Keuls multiple comparison test for more than three groups. All p values were two-tailed, with statistical significance set at p < 0.05.

## 3 Results

### 3.1 Pretreatment with high-dose AA leads to enhanced sensitivity to CD8+ T cell cytotoxicity in H460 cells

**Figure 1:**
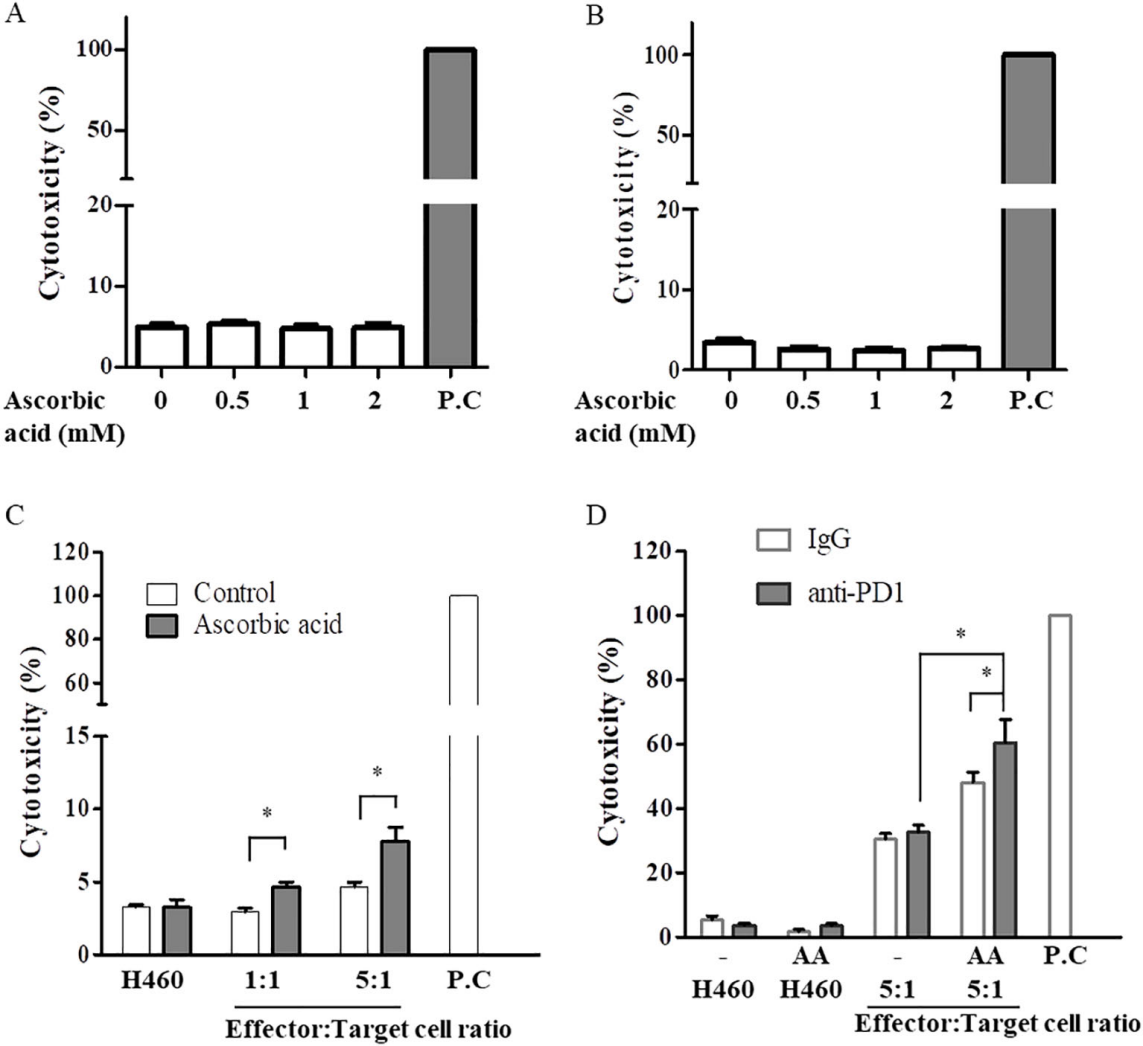
High-dose AA increased cytotoxic activity of CD8+ T cell for H460 cells. CD8+ T cells derived from healthy donor **(A)** or H460 cells **(B)** were pretreated with 100 µg/ml catalase for 30 min and then exposed to the indicated concentration of AA for 6h. Cells were resuspended in fresh medium and incubated for 48h. Cytotoxicity was measured using the LDH cytotoxicity assay kit. Positive control (P.C) was leading to 100% cytotoxicity by lysing the cells completely. **(C)** Activated CD8+ T cells (effector) by anti-CD3/CD28 beads or H460 cells (target) were pretreated with catalase and AA for 6 h, resuspended in fresh medium. AA-pretreated two cell lines (effector and target cells) were cocultured for 24 h at 1:1 or 5:1, ratios of effector:target cells. Data are presented as the mean ± SD of three experiments (*p < 0.05). **(D)** AA-pretreated two cell lines (effector and target cells) were cocultured with 5 µg/ml anti-PD1 for 48 h at a ratio of 5 (effector):1(target). Data are presented as the mean ± SD of six experiments (*p < 0.05).

To select lung cancer cell lines that could respond well to PD1, the expression levels of PD-L1 mRNA were tested by real-time PCR in three human NSCLC cells lines: H460, H1299, and A549. The highest PD-L1 mRNA expression was observed in H460 cells, which may be a good target for anti-PD1 treatment (**Supplementary Figure S1**). To test the cytotoxic effects of T cells, CD8+ T cells were isolated from healthy donors. Because the *in vitro* conditions did not have an antioxidant mechanism, CD8+ T cells and H460 cells were pretreated with 1000 unit/ml catalase for 30 min. The cells were treated with 1 mM AA for 6 h and then incubated in fresh medium for 48 h. As shown in **Figures 1A, B**, AA did not significantly affect the death of CD8+ T cells or H460 cells after 48 h at the indicated concentrations. CD8+ T cells and H460 cells pretreated with AA and catalase for 6 h were co-cultured with fresh media at different ratios of effector (T cells) to target (H460 cells) cells. High-dose AA significantly increased CD8+ T cell activity, as evidenced by increased cytotoxicity when cocultured at both 1 versus 1 and 5 versus 1, effector versus target cells ratios (**Figure 1C**). Next, we tested the effects of the combination of anti-PD1 and high-dose AA. Treatment with anti-PD1 alone did not significantly affect the cytotoxicity; however, treatment with a high dose of AA increased the cytotoxicity (**Figure 1D**).

### 3.2 Treatment with high-dose AA induces synergic effect with anti-PD1 treatment in a syngeneic lung cancer mouse model

**Figure 2:**
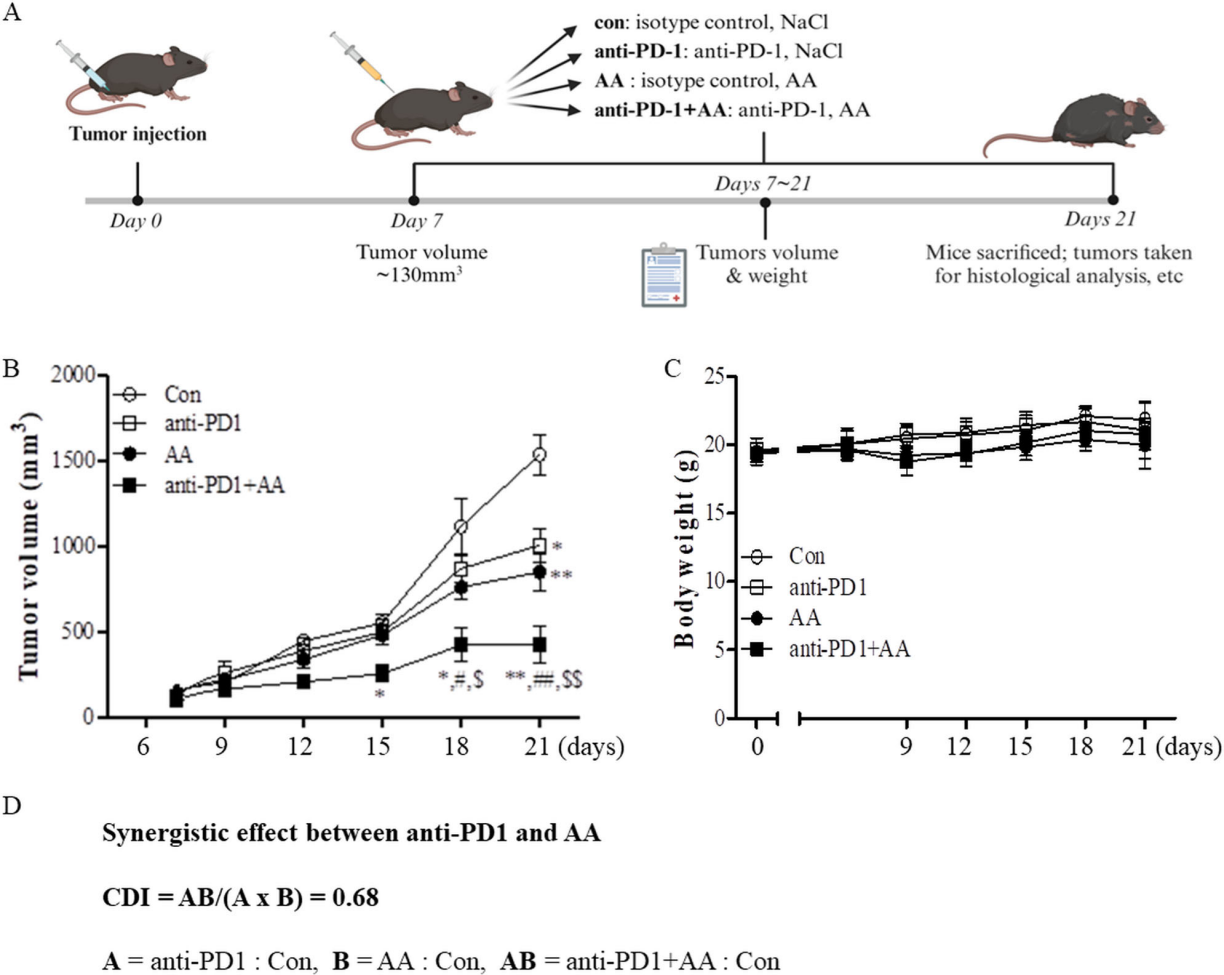
The *in vivo* antitumor efficacy of high-dose AA alone and in combination with anti-PD1 **(A)** Schematic workflow of the study in syngeneic tumor model to examine the synergic effects between anti-PD1 treatment and high-dose AA: C57BL/6J mice were inoculated subcutaneously into the right abdominal region with 1x10^6^ LLC1 mouse lung cancer cells in matrigel. Tumor-bearing mice were randomly assigned to four groups: vehicle plus isotype control (Con, n=5), vehicle plus anti-PD1 (anti-PD1, n=5), AA plus isotype control (AA, n=6), and AA plus anti-PD1 (AA+ anti-PD1, n=6). After 7 days, when the tumor masses reached ~130 mm^3^, 300 μL injections of 1.5 M AA were given daily and 200 μg of anti-PD1 was administered every other day. **(B)** Tumor volumes were observed every three days in accordance with the formula (*L* х *l* ^2^)/2. Significant differences were analyzed using two-way ANOVA test with the Bonferroni *post-hoc* tests. * and ** indicate p<0.05 and p<0.01, respectively, compared to control group (con). # and ## indicate p<0.05 and p<0.01, respectively, compared to AA. *$* and *$$* indicate p<0.05 and p<0.01, respectively, compared to anti-PD1. **(C)** Body weights were analyzed in the four groups of mice. **(D)** CDI was calculated to test for synergistic effect between anti-PD1 and AA. The CDI of 0.68 indicates synergy (defined as CD < 0.7 indicating a significantly synergistic effect).

We evaluated the effects of AA in an LLC1 lung cancer syngeneic mouse model to confirm the results in the co-culture model of H460 and CD8^+^ T cells. Tumor-bearing mice were treated with vehicle, anti-PD1, high-dose AA, or co-treated with anti-PD1 and high-dose AA when the tumor volume increased to ~130 mm^3^ until day 7. The mice were then sacrificed on day 21 (**Figure 2A**). As shown in **Figure 2B**, treatment with anti-PD1 or high-dose AA did not significantly affect tumor growth until day 15, but tended to slightly reduce tumor growth after day 18 compared to the vehicle treatment group. However, when these two drugs were administered in combination, significant antitumor effects were observed, and tumor growth was considerably reduced compared with treatment with anti-PD1 or high-dose AA (**Figure 2B**). For all experimental periods, although a slight weight loss was observed on day 9 in the AA-treated group, the body weight of the mice was not significantly affected (**Figure 2C**). Next, we tested the coefficient of drug interaction (CDI) formula to calculate the synergistic effect. The value of CDI between high-dose AA and anti-PD1 was 0.68, which indicated that high-dose AA exerted a significant synergistic effect with anti-PD1 treatment (**Figure 2D**). Collectively, our data indicate that high-dose AA could affect the sensitization of antitumor effects of anti-PD1 in both *in vitro* and *in vivo* models.

### 3.3 High-dose AA and anti-PD1 cotreatment leads to increased CD8+ T cell-dependent cytotoxicity

**Figure 3:**
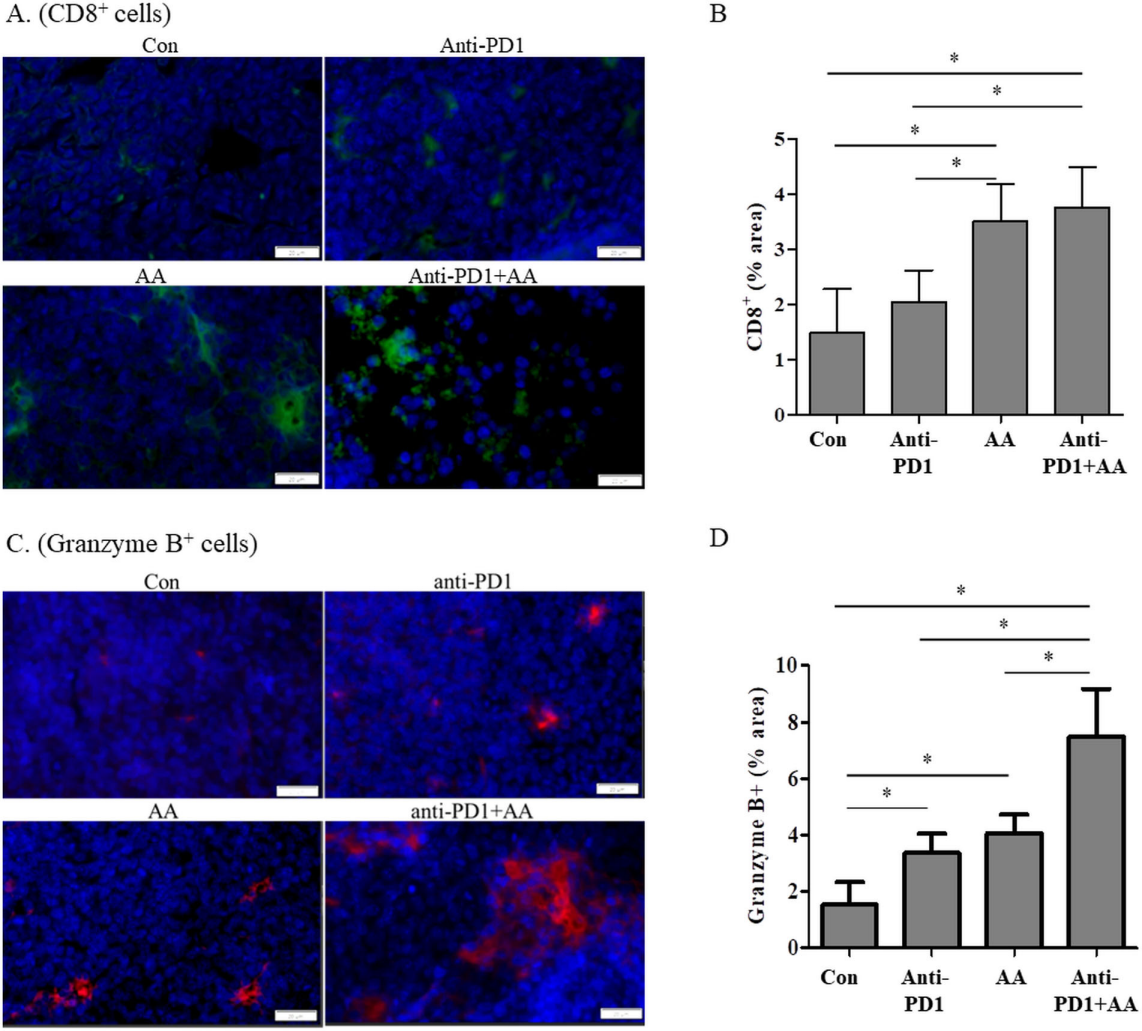
The combination of anti-PD1 and high-dose AA increase the CD8+ T cell infiltration and Granzyme B production in tumor tissues from syngeneic mouse model. **(A)** Tumor sections from syngeneic mouse model were stained with CD8 and immunofluorescence images were captured at 100x magnification. **(B)** % area was quantified by image J software. **(C)** Tumor sections from syngeneic mouse model were stained with Granzyme B and immunofluorescence images were captured at 100x magnification. **(D)** % area was quantified by image J software. Statistical analysis between four groups was evaluated using one-way ANOVA with the Newman-Keuls multiple comparison test. * indicates p < 0.05.

We hypothesized that high-dose AA and anti-PD1 cotreatment increased CD8+ T cells and changed the tumor microenvironment; to test this hypothesis, we used immunofluorescence to investigate markers of immune cell infiltration and function in a syngeneic lung tumor. High-dose AA treatment resulted in increased immunofluorescence of CD8, irrespective of anti-PD1 treatment in the tumor tissues (**Figures 3A, B**). CD8 + T infiltration was significantly higher in the AA-treated group than in the control and anti-PD1 groups. The infiltration of CD8+ T cells in the high-dose AA and anti-PD1 co-treatment group was not significantly different from that in the AA alone group. The cytotoxic effects of CD8+ T cells were assessed by granzyme B staining. The number of granzyme B-positive cells in the tissues of the AA-alone or anti-PD1 treatment groups was higher than that in the control group (**Figures 3C, D**). In the tumor tissues of the AA and anti-PD1 co-treatment group, the number of granzyme B-positive cells was significantly higher than that in the AA alone or anti-PD1 treatment groups (**Figures 3C, D**). Our data indicated that high-dose AA increased the infiltration of CD8+ T cells independently of anti-PD1, but increased the cytotoxic activity of anti-PD1.

**Figure 4:**
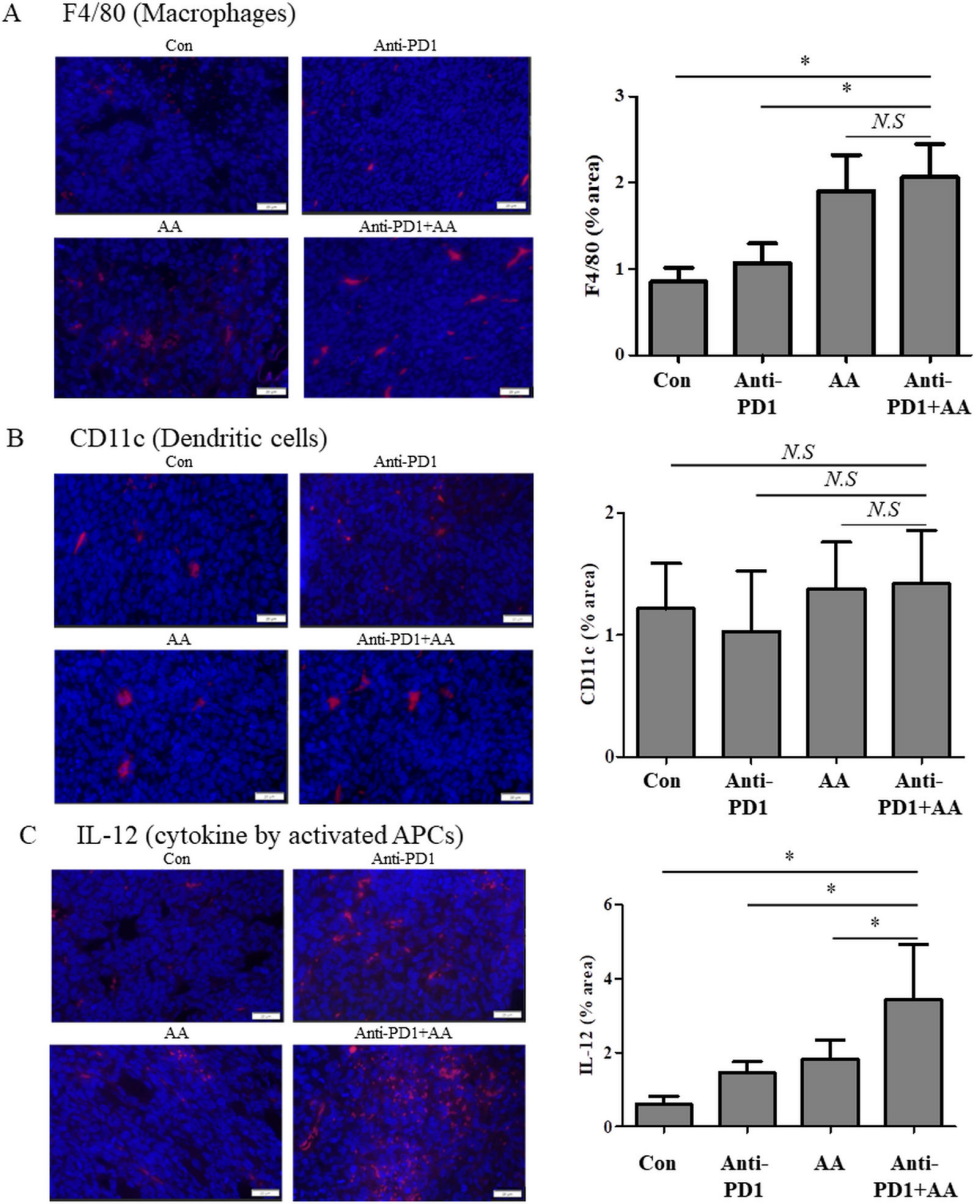
The combination of anti-PD1 and high-dose AA leads to increase the macrophage infiltration and IL-2 production in tumor tissues from syngeneic mouse model. Tumor sections from syngeneic mouse model were stained with **(A)** F4/80 (macrophages), **(B)** CD11c (dendritic cells) and **(C)** IL-12 (cytokine by activated APCs). Immunofluorescence images were captured at 100x magnification and quantified by image J software. Statistical analysis between four groups was evaluated using one-way ANOVA with the Newman-Keuls multiple comparison test. * indicates p < 0.05 and *N.S* indicates not significant.

We next investigated whether co-treatment with AA and anti-PD1 altered the infiltration of antigen-presenting cells (APCs) including macrophages (F4/80) and dendritic cells (CD11c), and enhanced the production of IL-2 by APCs. High-dose AA treatment resulted in increased infiltration of macrophages (F4/80), irrespective of anti-PD1 treatment in the tumor tissue (**Figure 4A**). However, the infiltration of dendritic cells (CD11c) did not differ significantly between the groups (**Figure 4B**). In addition, the production of IL-12 by APCs in the tissues of the anti-PD1 treatment group was higher than that in the control group, and the production of IL-12 in the tumor tissues of the AA and anti-PD1 co-treatment group was significantly higher than that in the anti-PD1 treatment group (**Figure 4C**). This indicates that high-dose AA and anti-PD1 co-treatment increased macrophage infiltration and activity.

### 3.4 Proteomic analysis of the synergic effects of high-dose AA in anti-PD1-treated syngeneic tumor

**Figure 5:**
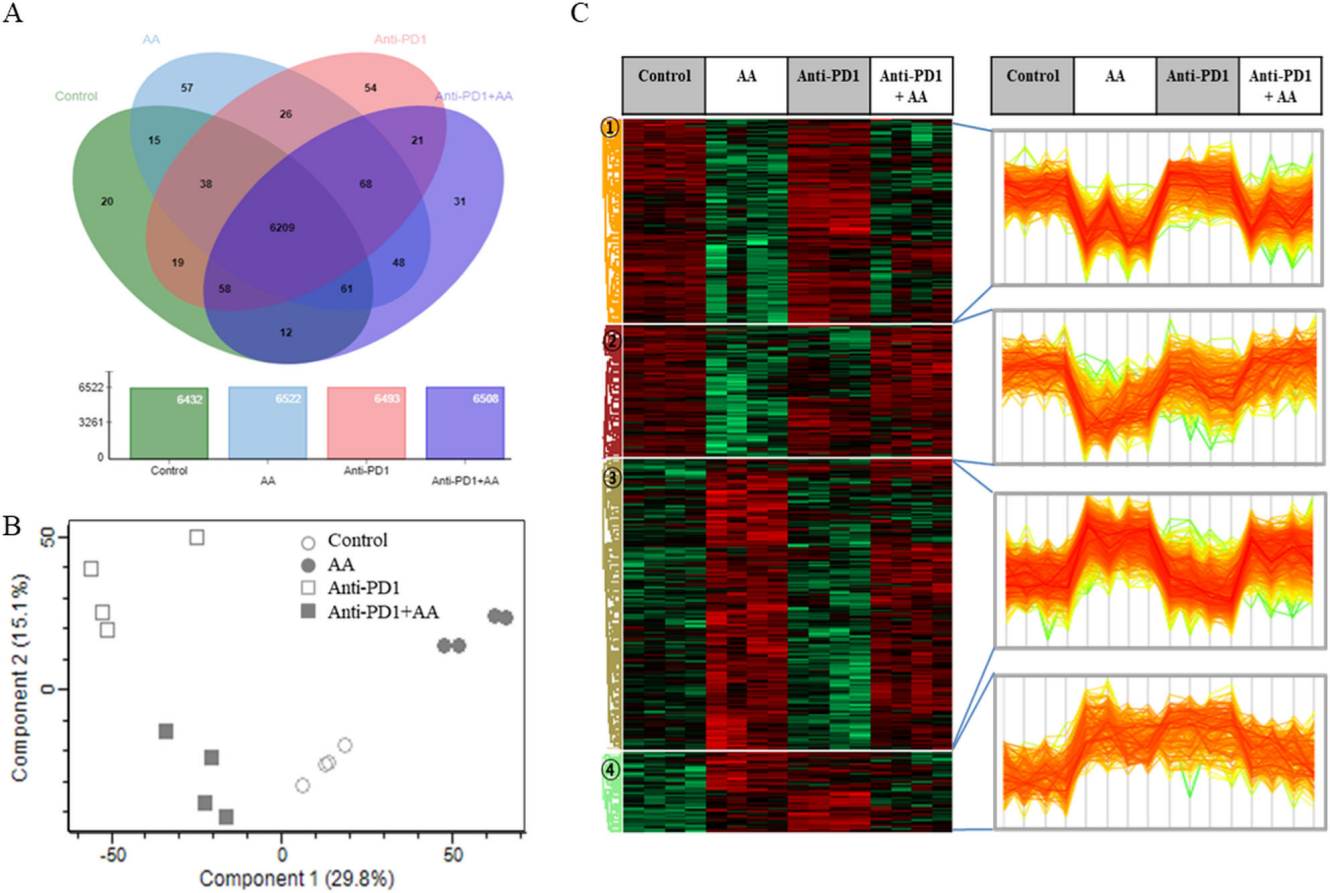
Proteomic analysis of the combination effects of high-dose AA and anti-PD1 in tumor tissues form syngeneic mouse model. **(A)** Venn diagram depicting the abundance of proteins among the four groups. **(B)** The principal component analysis (PCA) results for the four groups show that they are well separated. **(C)** Differentially expressed proteins (ANOVA, Benjamini-Hochberg method-based FDR < 0.05) were obtained via Z-score normalization and visualized using a heatmap. Proteins were grouped into four clusters (distance threshold =2.183). Clusters 1 and 2 were proteins down-regulated by AA and clusters 3 and 4 were proteins up-regulated by genistein compared to controls. In anti-PD1 treated groups, Clusters 1 and 3 were proteins affected by AA and were either down-regulated or up-regulated by AA, respectively, compared to treatment of anti-PD1 alone but cluster 2 and 4 did not significant affect by AA.

**Figure 6:**
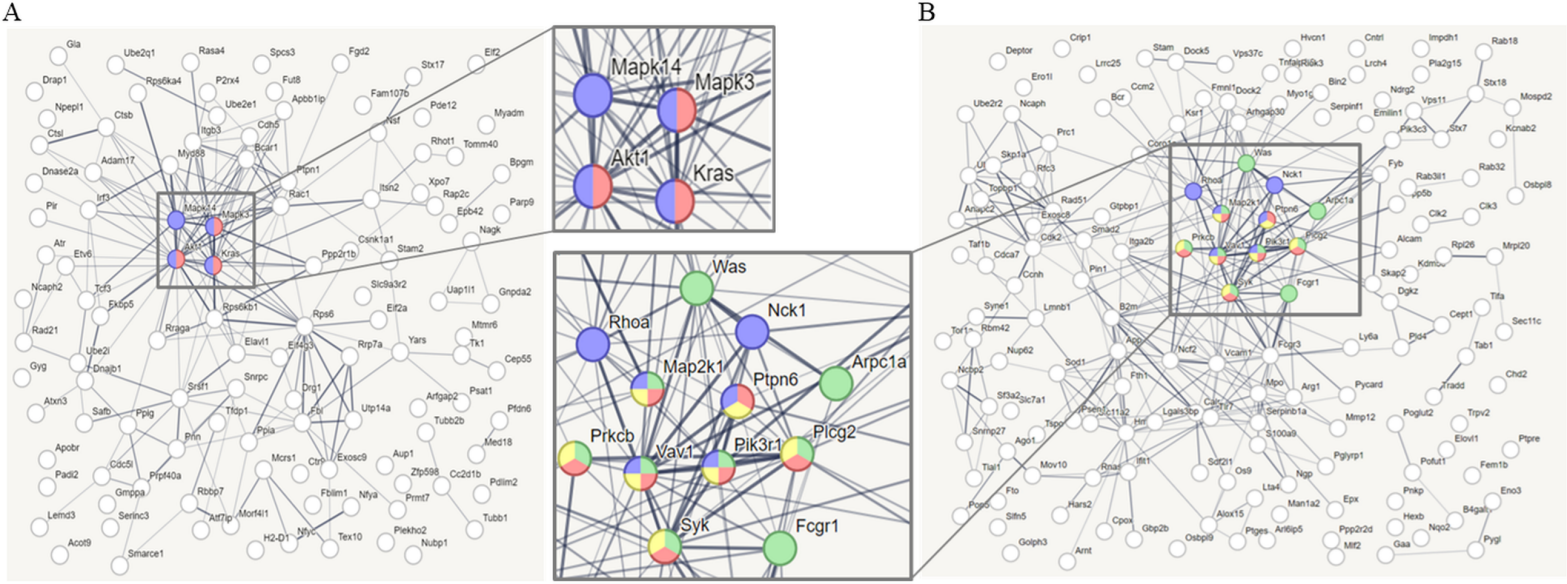
Protein-protein interaction (PPI) analysis **(A)** PPI network analyzed through STRING web site (www.String-db.org, PPI enrichment p < 7.74e-7), using as input data that immune system-related genes (90 genes, BTO:0005810) among the differentially expressed genes (DEGs, 442 genes) in cluster 1. Red nodes (3 genes) denote proteins implicated in the B cell receptor signaling pathway (KEGG pathways, mmu04662), and blue nodes (4 genes) denote proteins implicated in the T cell receptor signaling pathway (KEGG pathways, mmu04660). **(B)** PPI network analyzed through STRING web site (PPI enrichment p < 1.0e-16), using as input data that immune system-related genes (124 genes, BTO:0005810) among the DEGs (615 genes) in cluster 3. Red nodes (6 genes) denote proteins implicated in the B cell receptor signaling pathway (KEGG pathways, mmu04662), blue nodes (7 genes) denote proteins implicated in the T cell receptor signaling pathway (KEGG pathways, mmu04660), green nodes (9 genes) denote proteins implicated in the Fc gamma R-mediated phagocytosis (KEGG pathways, mmu04666), and yellow nodes (7 genes) denote proteins implicated in Natural killer (NK) cell mediated cytotoxicity (KEGG pathways, mmu04650). Line thickness indicated the strength of data support.

To confirm the synergistic effects of high-dose AA and anti-PD1 co-treatment in a syngeneic mouse model, we performed proteomic profiling using nano-LC-ESI-MS/MS. Data of 6737 proteins were obtained from four groups (control, AA, anti-PD1, and AA+anti-PD1), and 6209 proteins which were identified in all four groups were considered for further analysis (**Figure 5A**; **Supplementary Table S1**). Principal component analysis showed that the four groups were well-separated (**Figure 5B**). Next, 1533 proteins were selected using one-way ANOVA (Benjamini-Hochberg method-based FDR < 0.05). Using 1533 proteins, hierarchical clustering (distance threshold = 2.18) was performed and four clusters were generated (**Figure 5C**). Clusters 1 and 2 were downregulated by AA, and clusters 3 and 4 were upregulated by genistein compared with those in the controls. In the anti-PD1 treated groups, clusters 1 and 3 were proteins affected by AA and were either downregulated or upregulated by AA or co-treatment with AA and anti-PD1, respectively, compared to anti-PD1 treatment alone; however, proteins in clusters 2 and 4 were not significantly affected by co-treatment with AA and anti-PD1. Therefore, we conducted further studies focusing on the proteins (1066 proteins) in clusters 1 and 3, which may serve as therapeutic targets for co-treatment with anti-PD1 and AA. Protein-protein interaction (PPI) network analysis was performed using the STRING website (www.String-db.org) and 226 immune system-related proteins (BTO:0005810), including 90 proteins from Cluster 1 (**Supplementary Table S2**) and 124 proteins from Cluster 3 (**Supplementary Table S3**) (**Figure 6**). The PPI network using 90 proteins downregulated by co-treatment with anti-PD1 and AA as input showed three proteins (KRAS, MAPK3, and AKT1) for the B-cell receptor signaling pathway and four proteins (MAPK14, KRAS, MAPK3, and AKT1) for the T-cell receptor signaling pathway (**Figure 6A**). Among the 124 proteins upregulated by co-treatment with anti-PD1 and AA, the PPI network showed that 12 proteins interacted with each other to upregulate B (six proteins) and T cell receptor signaling pathways (seven proteins), Fc gamma R-mediated phagocytosis (nine proteins), and natural killer (NK) cell-mediated cytotoxicity (seven proteins) (**Figure 6B**).

## 4 Discussion

In this study, we investigated whether high-dose AA in combination with anti-PD1 therapy exhibits anticancer effects *in vitro* and in a syngeneic lung cancer mouse model, and determined the changes in the immune microenvironment of the tumor tissue. Our data showed that pretreatment with a high dose of AA enhanced the sensitivity to anti-PD1-treated CD8+ T cell cytotoxicity in H460 cells *in vitro*. Second, the investigation of tumor volume and CDI value (0.68) in a syngeneic lung cancer mouse model showed that high-dose AA could act as a promising agent to enhance the antitumor effects of anti-PD1 therapy. Third, the combination of anti-PD1 and high-dose AA increased immune activity by promoting granzyme B and IL-12 production by T cells and macrophages, respectively. Finally, the antitumor mechanisms of high-dose AA in combination with anti-PD1 were comprehensively investigated through proteomic profiling of the tumor tissue of a mouse model, which indicated that high-dose AA exerted antitumor effects by regulating various immune-related mechanisms, including the B cell and T cell receptor signaling pathways, Fc gamma R-mediated phagocytosis, and NK cell-mediated cytotoxicity.

ICIs have become the mainstay of therapy for patients with NSCLC without driver mutations. However, durable response remains limited to a small proportion of patients (20–30%) (2). To overcome these limitations of ICIs, efforts are ongoing to improve the efficacy of these drug by combination with potentiating agents (23). In our study, we investigated the combined effect of anti-PD1 and high-dose AA and showed that these two drugs had synergistic effects in a lung cancer mouse model. Because anti-PD1 is an immunomodulator, we first performed a PPI analysis focusing on the changes in immune system-related proteins caused by the combined effect of anti-PD1 and high-dose AA in tumor tissues from a syngeneic lung cancer mouse model. The four immune system-related proteins (Akt1, Mapk14, Mapk3, and Kras) that were reduced by the combination of anti-PD1 antibody and high-dose AA were AKT and MAPK signaling proteins, which are involved in cell growth (24–26). The Immune system-related proteins increased by the combination of anti-PD1 and high-dose AA were involved in B cell and T cell receptor signaling pathways, Fc gamma R-mediated phagocytosis, and NK cell-mediated cytotoxicity. In previous studies, AA treatment has been shown to increase the activity of major immune cells (T cells, dendritic cells, macrophages, and NK cells) (14, 17, 27). Moreover, the synergistic effect of these two drugs in our study was additionally supported by increased granzyme B production by cytotoxic T cells and increased IL-12 production by APCs. Our results suggest that the combination of anti-PD1 and high-dose AA may act synergistically by modulating the activity of cytotoxic T cells and other immune cells, such as NK cells and APCs.

Increasing evidence suggests that high-dose intravenous AA is a promising anticancer agent. Many clinical trials on high-dose AA have confirmed its safety and efficacy in eradicating cancer cells in various malignancies through various mechanisms (17, 23, 28). The anticancer effects of high-dose AA via multi-targeting have been demonstrated, which include prooxidative cytotoxic agent, anticancer epigenetic regulator, and immune modulator. In addition, high-dose AA inhibited epithelial-to-mesenchymal transition, hypoxia, and oncogenic kinase signaling (23, 29). Moreover, high-dose AA is safe, tolerable, and potentially efficacious for lung cancer treatment; it can act synergistically with chemotherapy and used as an agent for reducing the toxic side effects of cancer therapy (23, 29–31). The role of AA as a modulator of immune response has been reported in a few studies. AA modulates the function of several immune cells and may therefore have beneficial effects in cellular immunotherapy. Luchtel et al. demonstrated that high-dose AA had a significant synergistic effect with anti-PD1 therapy in a lymphoma mouse model (14). The results of this study showed that high-dose AA increased the intratumoral infiltration of CD4^+^ and CD8^+^ T cells and macrophages into the tumor microenvironment, with increased production of granzyme B and interleukin-12. Furthermore, high-dose AA has been shown to modulate the infiltration of immune cells into the tumor microenvironment, enhance the cytotoxic activity of adoptively transferred CD8 + T cells, and cooperate with anti-PD1 and anti-CTLA-4 treatments in mice with syngeneic tumors (15). Several previous studies have shown that AA acts as a cofactor for DNA and histone demethylases, thereby inducing the epigenetic regulation of T cell activation (27, 32).

However, the precise mechanism of action of high-dose AA as an adjuvant for anti-PD1 treatment in lung cancer is not yet completely understood. However, the mechanisms underlying the therapeutic effects of AA combined with ICI require further investigation. In this study, we showed *in vitro* that high-dose AA increases the cytotoxic activity of CD8+ T cells using anti-PD1. Proteomic analysis showed that high-dose AA is involved in the regulation of various immune cells in anti-PD1-treated tumor tissue in a syngeneic tumor model. These results suggest that high-dose AA enhances the therapeutic effect of anti-PD1 as an adjuvant in NSCLC.

This syngeneic lung cancer mouse model has some limitations. First, unlike humans, C57BL/6J mice can synthesize vitamin C. Therefore, it is necessary to study whether the cancer environment affects the synthesis of vitamin C in mice and whether the administration of high-dose AA exceeds that of AA synthesized by mice. However, our results showed that treatment of high-dose AA significantly reduced tumor growth and increased CD8 and F40/80 expression compared to vehicle-treated controls. Moreover, the administration of high-dose AA significantly increased the anticancer effect of anti-PD1, which is similar to the results of other studies (14). These data suggest that high-dose AA treatment may induce AA levels beyond those endogenously synthesized in mice, potentially creating a more permissive microenvironment for anticancer immune responses. Second, the number of mice in each group was too small to accurately evaluate the combined effects of high-dose AA and anti-PD1. Nevertheless, we verified the role of high-dose AA on the anticancer efficacy of anti-PD1 using an *in vitro* model. Finally, mouse cancer models are unlikely to accurately represent the clinical state of lung cancer. Immunotherapy is administered to patients with established lung cancer. In our model, high-dose AA was administered 7 days after transplantation and the growth rate was monitored. Whether 7 days is sufficient for tumor establishment should be considered. Therefore, further studies are needed to control for the period of tumor establishment.

In conclusion, our results highlight the important role of high-dose AA in the anticancer efficacy of anti-PD1 in lung cancer models and suggest that high-dose AA may be a potential adjuvant for improving the efficacy of anti-PD1 immunotherapy in NSCLC. The protein profiling data provide comprehensive information for further studies to understand the molecular mechanism of high-dose AA in anti-PD1-treated lung cancer. Furthermore, our study provides a rational preclinical basis to test the combination therapy of anti-PD1 plus high-dose AA in lung cancer and paves the way for testing promising combinations of the two drugs in preclinical studies of other cancer types.

## Data Availability

The data presented in the study are deposited in the ProteomeXchange repository, accession number: PDX059688.

## References

[1] Siegel RL Miller KD Fuchs HE Jemal A. Cancer statistics, 2022. CA Cancer J Clin. (2022) 72:7–33. doi: 10.3322/caac.21708 35020204

[2] Ribas A Wolchok JD. Cancer immunotherapy using checkpoint blockade. Science. (2018) 359:1350–5. doi: 10.1126/science.aar4060 PMC7391259 29567705

[3] Burns JJ. Missing step in man, monkey and Guinea pig required for the biosynthesis of L-ascorbic acid. Nature. (1957) 180:553. doi: 10.1038/180553a0 13477232

[4] Nishikimi M Fukuyama R Minoshima S Shimizu N Yagi K. Cloning and chromosomal mapping of the human nonfunctional gene for L-gulono-gamma-lactone oxidase, the enzyme for L-ascorbic acid biosynthesis missing in man. J Biol Chem. (1994) 269:13685–8. doi: 10.1016/S0021-9258(17)36884-9 8175804

[5] Carr AC Maggini S. Vitamin C and immune function. Nutrients. (2017) 9(11):1211. doi: 10.3390/nu9111211 PMC5707683 29099763

[6] Cameron E Pauling L. Supplemental ascorbate in the supportive treatment of cancer: reevaluation of prolongation of survival times in terminal human cancer. Proc Natl Acad Sci U.S.A. (1978) 75:4538–42. doi: 10.1073/pnas.75.9.4538 PMC336151 279931

[7] Creagan ET Moertel CG O’Fallon JR Schutt AJ O’Connell MJ Rubin J. Failure of high-dose vitamin C (Ascorbic acid) therapy to benefit patients with advanced cancer. A controlled trial. N Engl J Med. (1979) 301:687–90. doi: 10.1056/NEJM197909273011303 384241

[8] Moertel CG Fleming TR Creagan ET Rubin J O’Connell MJ Ames MM. High-dose vitamin C versus placebo in the treatment of patients with advanced cancer who have had no prior chemotherapy. A randomized double-blind comparison. N Engl J Med. (1985) 312:137–41. doi: 10.1056/NEJM198501173120301 3880867

[9] Huijskens MJ Wodzig WK Walczak M Germeraad WT Bos GM. Ascorbic acid serum levels are reduced in patients with hematological Malignancies. Results Immunol. (2016) 6:8–10. doi: 10.1016/j.rinim.2016.01.001 27014565 PMC4792862

[10] Mayland CR Bennett MI Allan K. Vitamin C deficiency in cancer patients. Palliat Med. (2005) 19:17–20. doi: 10.1191/0269216305pm970oa 15690864

[11] White R Nonis M Pearson JF Burgess E Morrin HR Pullar JM. Low vitamin C status in patients with cancer is associated with patient and tumor characteristics. Nutrients. (2020) 12(8):2338. doi: 10.3390/nu12082338 PMC7468872 32764253

[12] Huijskens MJ Walczak M Koller N Briede JJ Senden-Gijsbers BL Schnijderberg MC. Technical advance: ascorbic acid induces development of double-positive T cells from human hematopoietic stem cells in the absence of stromal cells. J Leukoc Biol. (2014) 96:1165–75. doi: 10.1189/jlb.1TA0214-121RR 25157026

[13] Manning J Mitchell B Appadurai DA Shakya A Pierce LJ Wang H. Vitamin C promotes maturation of T-cells. Antioxid Redox Signal. (2013) 19:2054–67. doi: 10.1089/ars.2012.4988 PMC3869442 23249337

[14] Luchtel RA Bhagat T Pradhan K Jacobs WR Jr. Levine M Verma A. High-dose ascorbic acid synergizes with anti-pd1 in a lymphoma mouse model. Proc Natl Acad Sci U.S.A. (2020) 117:1666–77. doi: 10.1073/pnas.1908158117 PMC6983418 31911474

[15] Magri A Germano G Lorenzato A Lamba S Chila R Montone M. High-dose vitamin C enhances cancer immunotherapy. Sci Transl Med. (2020) 12:eaay8707. doi: 10.1126/scitranslmed.aay8707 32102933

[16] Zhao X Liu M Li C Liu X Zhao J Ma H. High dose vitamin C inhibits pd-L1 by ros-pstat3 signal pathway and enhances T cell function in tnbc. Int Immunopharmacol. (2024) 126:111321. doi: 10.1016/j.intimp.2023.111321 38041955

[17] Ang A Pullar JM Currie MJ Vissers MCM. Vitamin C and immune cell function in inflammation and cancer. Biochem Soc Trans. (2018) 46:1147–59. doi: 10.1042/BST20180169 PMC6195639 30301842

[18] Chen Q Espey MG Krishna MC Mitchell JB Corpe CP Buettner GR. Pharmacologic ascorbic acid concentrations selectively kill cancer cells: action as a pro-drug to deliver hydrogen peroxide to tissues. Proc Natl Acad Sci U.S.A. (2005) 102:13604–9. doi: 10.1073/pnas.0506390102 PMC1224653 16157892

[19] Chen Q Espey MG Sun AY Pooput C Kirk KL Krishna MC. Pharmacologic doses of ascorbate act as a prooxidant and decrease growth of aggressive tumor xenografts in mice. Proc Natl Acad Sci U.S.A. (2008) 105:11105–9. doi: 10.1073/pnas.0804226105 PMC2516281 18678913

[20] Klingelhoeffer C Kammerer U Koospal M Muhling B Schneider M Kapp M. Natural resistance to ascorbic acid induced oxidative stress is mainly mediated by catalase activity in human cancer cells and catalase-silencing sensitizes to oxidative stress. BMC Complement Altern Med. (2012) 12:61. doi: 10.1186/1472-6882-12-61 22551313 PMC3404974

[21] Kim S Nam Y Kim MJ Kwon SH Jeon J Shin SJ. Proteomic analysis for the effects of non-saponin fraction with rich polysaccharide from korean red ginseng on alzheimer’s disease in a mouse model. J Ginseng Res. (2023) 47:302–10. doi: 10.1016/j.jgr.2022.09.008 PMC10014184 36926613

[22] Kwon SH Chung H Seo JW Kim HS. Genistein alleviates pulmonary fibrosis by inactivating lung fibroblasts. BMB Rep. (2024) 57:143–8. doi: 10.5483/BMBRep.2023-0099 PMC10979345 37817434

[23] Bottger F Valles-Marti A Cahn L Jimenez CR. High-dose intravenous vitamin C, a promising multi-targeting agent in the treatment of cancer. J Exp Clin Cancer Res. (2021) 40:343. doi: 10.1186/s13046-021-02134-y 34717701 PMC8557029

[24] Tsai PJ Lai YH Manne RK Tsai YS Sarbassov D Lin HK. Akt: A key transducer in cancer. J BioMed Sci. (2022) 29:76. doi: 10.1186/s12929-022-00860-9 36180910 PMC9526305

[25] Singhal A Li BT O’Reilly EM. Targeting kras in cancer. Nat Med. (2024) 30:969–83. doi: 10.1038/s41591-024-02903-0 PMC11845254 38637634

[26] Bahar ME Kim HJ Kim DR. Targeting the ras/raf/mapk pathway for cancer therapy: from mechanism to clinical studies. Signal Transduct Target Ther. (2023) 8:455. doi: 10.1038/s41392-023-01705-z 38105263 PMC10725898

[27] Jeong YJ Kim JH Hong JM Kang JS Kim HR Lee WJ. Vitamin C treatment of mouse bone marrow-derived dendritic cells enhanced cd8(+) memory T cell production capacity of these cells *in vivo* . Immunobiology. (2014) 219:554–64. doi: 10.1016/j.imbio.2014.03.006 24698552

[28] Cameron E Pauling L. Supplemental ascorbate in the supportive treatment of cancer: prolongation of survival times in terminal human cancer. Proc Natl Acad Sci U.S.A. (1976) 73:3685–9. doi: 10.1073/pnas.73.10.3685 PMC431183 1068480

[29] Schoenfeld JD Sibenaller ZA Mapuskar KA Wagner BA Cramer-Morales KL Furqan M. O(2)(·-) and H(2)O(2)-mediated disruption of fe metabolism causes the differential susceptibility of nsclc and gbm cancer cells to pharmacological ascorbate. Cancer Cell. (2017) 32:268. doi: 10.1016/j.ccell.2017.07.008 28810149

[30] Lee KE Hahm E Bae S Kang JS Lee WJ. The enhanced tumor inhibitory effects of gefitinib and L-ascorbic acid combination therapy in non-small cell lung cancer cells. Oncol Lett. (2017) 14:276–82. doi: 10.3892/ol.2017.6109 PMC5494887 28693165

[31] Furqan M Abu-Hejleh T Stephens LM Hartwig SM Mott SL Pulliam CF. Pharmacological ascorbate improves the response to platinum-based chemotherapy in advanced stage non-small cell lung cancer. Redox Biol. (2022) 53:102318. doi: 10.1016/j.redox.2022.102318 35525024 PMC9079696

[32] Young JI Zuchner S Wang G. Regulation of the epigenome by vitamin C. Annu Rev Nutr. (2015) 35:545–64. doi: 10.1146/annurev-nutr-071714-034228 PMC4506708 25974700

