# Supplementary material for: High-dose ascorbic acid synergizes with anti-PD1 therapy in non-small cell lung cancer in vitro and in vivo models
Source: Front Immunol. 2025 Jan 17;15:1512605. doi: 10.3389/fimmu.2024.1512605 (PMC11783322; doi:10.3389/fimmu.2024.1512605)
Supplement: Supplementary file 1 [file DataSheet1.docx]

High-dose ascorbic acid synergizes with anti-PD1 therapy in non-small cell lung cancer cells

**Hak Su Kim^1†^, Seung-hyun Kwon^1^****^†^, Ok Kyung Choi^1^, and Taekyu Lim*,^1,2^**

^1^Veterans Medical Research Institute, Veterans Health Service Medical Center, Seoul 05368, Republic of Korea

^2^Division of Hematology-Oncology, Department of Internal Medicine, Veterans Health Service Medical Center, Seoul 05368, Republic of Korea

**^†^**These authors equally contribute to this work

***Correspondence:**Taekyu Lim**,** Division of Hematology-Oncology, Department of Internal Medicine, Veterans Health Service Medical Center, Seoul 05368, Republic of Korea 53, Jinhwangdo-ro 61-gil, Gangdong-gu, Seoul 05368, Republic of Korea, Tel: +82-2-2225-1492, Fax: +82-2-2225-3950, E-mail: imegene@naver.com

**Supplementary Materials and methods.**

**Cell culture**

Human NSCLC cell lines including H1299 (ATCC), and A549 (ATCC) were cultured in RPMI1640 (Hyclone) supplemented with 10%FBS and 1% penicillin/streptomycin. Most cells were grown until approximately 70% confluence in culture dishes at 37°C in a 5% CO_2_ incubator and detached using 0.25% trypsin EDTA for passaging or use.

**RNA extraction and reverse transcription PCR**

Total RNA was extracted from the cells using TRIzol reagent (Invitrogen, Carlsbad, CA) based on the manufacturer’s instructions and was reverse transcribed to complementary DNA using M-MLV reverse transcriptase (Promega, Madison, WI) and oligo (dT) primers. cDNA were analyzed using real-time PCR. The sequences of the gene-specific primers are as follows: human PDL1 forward primer 5'-TGCCGACTACAAGCGAATTACTG-3' reverse primer 5'-CTGCTTGTCCAGATGACTTCGG-3' GAPDH forward primer 5'-CCCACTCCTCCACCTTTGAC-3', reverse primer 5'-TTGCTGTAGCCAAATTCGTTGT-3'

**Supplementary Figure**

Supplementary Figure S1. The mRNA expression of PD-L1 in H460, H1299 and A549 cells.

H460 cells showed the highest PD-L1 expression, while H1299 and A549 cells showed relatively low PD-L1 expression.
